# Plantamajoside Promotes NGF/TrkA Pathway to Inhibit Neuronal Apoptosis and Improve Diabetic Peripheral Neuropathy

**DOI:** 10.1111/jcmm.70571

**Published:** 2025-04-28

**Authors:** Qingshan Hai, Yuming Wang, Hanzhou Li, Huan Pei, Ning Wang, Xiaoxia Zhang, Mingyao Fan, Jiabao Liao, Weibo Wen, Jie Zhao, Ling Yang, Huantian Cui

**Affiliations:** ^1^ Nanjing University of Chinese Medicine Nanjin China; ^2^ Basic Medical School Yunnan University of Chinese Medicine Kunming China; ^3^ Tianjin University of Traditional Chinese Medicine Tianjin China; ^4^ First School of Clinical Medicine Yunnan University of Chinese Medicine Kunming China; ^5^ School of Nursing Yunnan University of Chinese Medicine Kunming China

**Keywords:** apoptosis, diabetic peripheral neuropathy, neurotrophin, NGF/TrkA pathway, Plantamajoside, transcriptomics

## Abstract

The symptoms caused by diabetic peripheral neuropathy (DPN) have severely impacted patients' quality of life. While plantamajoside (PMS) exhibits neuroprotective properties, its efficacy and molecular mechanisms against DPN are unexplored. This study first established a high glucose (HG)‐induced in vitro model of DPN and investigated the neuroprotective effects of PMS on RSC96 cells. We next demonstrated the anti‐apoptotic effects of PMS and NGF/TrkA pathway mediated neurotrophic effects. Finally, we established a DPN mouse model and confirmed the therapeutic effects of PMS on DPN mice through behavioural tests and pathological staining, while also assessing the impact of PMS on the NGF/TrkA pathway and apoptosis. Our results showed that, in HG‐induced DPN models, PMS enhanced cell viability while reducing LDH activity. Transcriptomics results indicated that the Apoptosis and Neurotrophins signalling pathways were key pathways for PMS on DPN. PMS treatment reduced HG‐induced RSC96 cell apoptosis while enhancing NGF levels and upregulating NGF/TrkA‐related protein expression. However, this protection was abolished by TrkA inhibitor or NGF neutralising antibodies. In vivo experimental results showed that PMS improved the mechanical pain threshold, thermal pain reaction time, and nerve conduction velocity of DPN mice. PMS improved pathological damage to the sciatic nerve, enhanced the number of Nissl bodies, reduced TUNEL‐positive expression, and upregulated NGF levels. Furthermore, PMS reduced apoptosis and elevated NGF/TrkA‐related protein expression in the sciatic nerve of DPN mice. In conclusion, PMS alleviates DPN through activating the NGF/TrkA pathway and inhibiting apoptosis.

**Abbreviations**
DM: diabetes mellitus
DPN: diabetic peripheral neuropathy
NGF: nerve growth factor
PMS: plantamajoside
HG: high glucose
STZ: streptozotocin
MNCV: motor nerve conduction velocity
SNCV: sensory nerve conduction velocity
DEGs: differentially expressed genes

## 1 Introduction

Diabetes Mellitus (DM) remains a major global public health challenge, with China accounting for 26.7% of the global diabetic population. According to the International Diabetes Federation (IDF) Diabetes Atlas 10th edition, approximately 141 million adults in China were living with DM, translating to an age‐adjusted prevalence rate of 10.6% [1]. Diabetic peripheral neuropathy (DPN) is a common chronic complication of DM, with reported incidence rates varying widely [2]. The risk of DPN increases with age, and the lifetime incidence may exceed 50%, with approximately 20% of diabetic patients experiencing painful DPN [3]. DPN is characterised by sensory abnormalities, numbness, and hyperalgesia, and is a significant contributor to diabetic foot ulcers, gangrene, and even amputation [4]. Currently, there are no specific drugs developed exclusively for the treatment of DPN, and the commonly used clinical treatment strategies aim to alleviate DPN symptoms through glycaemic control, nerve repair, reduction of oxidative stress, enhancement of microcirculation, and improvement of metabolic disorders [5]. Therefore, there is an urgent need to develop new strategies.

The aetiology and pathogenesis of DPN are complex, and apoptosis of peripheral nerve cells is an important factor contributing to its development [6]. Studies have indicated that although there may be no obvious symptoms in the early stages of diabetes, apoptosis of cells in the dorsal root ganglia and sciatic nerve has already occurred [7]. Under normal conditions, the peripheral nervous system is protected by myelin sheaths. However, in a hyperglycemic state, apoptosis of Schwann cells, which form myelin sheaths, leads to demyelination, triggering apoptosis of peripheral nerve cells, subsequently causing imbalance in neural homeostasis, and ultimately progressing to nerve degeneration and pain [8]. Another important factor considered to induce DPN is the deficiency of neurotrophic factors. Among them, nerve growth factor (NGF), brain‐derived neurotrophic factor, and insulin‐like growth factor are involved in the occurrence and development of DPN [9]. In particular, the lack of NGF can lead to decreased physiological function and self‐healing ability of nerve tissue [10]. By improving peripheral nerve cell apoptosis through various pathways, such as nerve nourishment [11], inhibition of mitochondrial apoptosis [12], and alleviation of endoplasmic reticulum apoptosis [13], DPN can be effectively improved.

Natural products have been widely demonstrated in laboratories to possess unique advantages in improving DPN. For instance, quercetin plays a pivotal role in neuroprotection in DPN [14], gastrodin can effectively alleviate neuronal apoptosis and neuroinflammation in DPN [15, 16], and harmaline stands out in enhancing brain‐derived neurotrophic factor [17]. Plantamajoside (PMS), one of the main active ingredients in the traditional Chinese medicine *Plantago asiatica* L., exhibits diverse effects including antibacterial, anti‐inflammatory, antioxidant, and anti‐apoptotic properties [18]. Studies have shown that PMS has demonstrated neuroprotective effects in laboratory settings on Parkinson's disease mouse models [19] and rats with acute spinal cord injury [19]. However, the therapeutic effects of PMS on DPN have not been discussed, and further mechanistic studies have yet to be explored. Therefore, we initiated a study on the treatment of DPN with PMS and its related mechanisms.

Here, we first established a high glucose‐induced RSC96 cell injury model and administered different concentrations of PMS for intervention. Based on confirming the effect of PMS in improving RSC96 cell injury, we employed transcriptomics technology to investigate the impact of PMS on gene expression in high glucose‐induced RSC96 cells. These genes were primarily enriched in the Apoptosis and Neurotrophin signalling pathway pathways. Subsequently, we conducted a series of verifications on the regulatory effects of PMS on the Apoptosis and NGF/TrkA pathways. Finally, we further confirmed the significant potential of PMS in improving DPN through in vivo experiments. This study provides a new perspective for improving DPN, and the preliminary exploration of the mechanisms underlying PMS's improvement of DPN lays a foundation for subsequent targeted research and drug development.

## 2 Materials and Methods

### 2.1 Cells, Animals and Reagents

The RSC96 cell line (CL‐0199) was purchased from Wuhan Procell Life Science & Technology Co. Ltd. (Wuhan, China). The experimental animals were 7‐week‐old healthy male C57BL/6 mice, which were raised in an SPF‐grade environment with free access to food and water. The experimental animals were purchased from SiPeiFu (Production Licence Number: SYXK(Beijing)2019–0030). Our animal experiments have been approved by the Ethics Committee of Yunnan University of Chinese Medicine (Approval Number: R‐062023G234). The kits and antibodies used in our study are included in the Supporting Information. The structural formulas of PMS, lipoic acid (LA), and streptozotocin (STZ) are shown in Figures S1–S3.

### 2.2 In Vitro Experiment

#### 2.2.1 Cell Culture and Grouping

RSC96 cells were cultured statically in a DMEM medium containing 10% fetal bovine serum and 1% penicillin–streptomycin antibiotic mixture, placed in a cell incubator at 37°C with 5% CO_2_ and saturated humidity. The morphology of cells in good growth condition should resemble neuron‐like adherent cells. When the colour of phenol red in the medium turns yellow, or when switching to another medium for modelling, the medium needs to be replaced. When the cell density reaches over 80%, the cells are digested with 0.25% trypsin and subcultured at a ratio of 1:2 or 1:3.

Cell Grouping: (1) Construction of an in vitro DPN cell model, where RSC96 cells were exposed to different concentration gradients of glucose (0, 50, 100, 200, 400 and 800 mM) for 24 h. (2) Investigating the effect of various concentration gradients of PMS on the viability of RSC96 cells, by treating RSC96 cells with PMS at concentrations of 0, 5, 12.5, 25, 50, 100 μM for 24 h. (3) Assessing the impact of PMS on high glucose‐induced RSC96 cell viability and lactate dehydrogenase (LDH) levels in the supernatant. The groups included: Control group, Control + H‐PMS (50 μM) group, HG group (400 mM), HG + L‐PMS (12.5 μM) group, HG + M‐PMS (25 μM) group, and HG + H‐PMS group, with each treatment applied to RSC96 cells for 24 h. (4) Transcriptomic analysis of RSC96 cells induced by high glucose and intervened with PMS. The groups were: Control group, HG group and HG + H‐PMS group. (5) Investigating the effect of PMS on high glucose‐induced RSC96 cells after inhibiting NGF and TrkA. The groups were: Control group, HG group, HG + H‐PMS group, HG + GW 441756 group, HG + H‐PMS + GW 441756 group, HG + NGF neutralising antibody (NGFnAb) group and HG + H‐PMS + NGFnAb group. The same volume of dimethylsulfoxide (DMSO) without PMS was treated to RSC96 cells as the corresponding vehicle control.

#### 2.2.2 MTT Assay

We treated plated RSC96 cells with different concentrations of PMS for 24 h. Following this, we used the MTT method to determine the relative cell viability.

#### 2.2.3 Transcriptomics

After collecting the cells, we performed transcriptomic sequencing based on prior studies [20]. Briefly, collect cells from each group, isolate total RNA, and assess the purity, concentration, and integrity of the RNA samples. Once the samples meet the quality standards, proceed with library preparation and sequencing using the Illumina platform. Utilise DESeq2 software to analyse differentially expressed genes (DEGs) between HG vs. Control and H‐PMS vs. HG. The screening criteria for differentially expressed genes (DEGs) are set as |Log_2_(FoldChange)| ≥ 1 and *p*adj ≤ 0.05.

#### 2.2.4 Apoptosis Detection

RSC96 cells were seeded at 5 in 6‐well plates and cultured for 24 h. After treatment for another 24 h, cells were collected, and FITC‐Annexin V and PI fluorescence intensities were measured by flow cytometry to assess apoptosis levels [21].

### 2.3 In Vivo Experiment

#### 2.3.1 Modelling, Grouping and Administration

After 1 week of adaptive feeding, 60 C57BL/6 mice were randomly and evenly divided into six groups: the Control group, the DPN group, the LA group, the low‐dose PMS group (L‐PMS), the medium‐dose PMS group (M‐PMS) and the high‐dose PMS group (H‐PMS). Except for the Control group, DPN mouse models were established in the other five groups using methods referenced from previous studies [22]. Briefly, the mice were injected with 150 mg/kg STZ into the abdominal cavity in a single dose, while the Control group received the same volume of solvent buffer. DPN mice were defined through behavioural tests 6 weeks after injection. Subsequently, a 4‐week drug intervention was administered, with the LA dose set at 60 mg/kg and the PMS doses set at 25, 50 and 100 mg/kg, respectively. The dosage of PMS was set according to previous studies [19, 23, 24].

#### 2.3.2 Behavioural Tests

We referred to the previous research methods [22, 25]. Mechanical Pain Threshold: Mice were acclimated on a metal mesh for 30 min. The plantar surface of the hind paw was stimulated with increasing pressure using an electronic Von Frey tester. The pressure at paw withdrawal was recorded as the mechanical pain threshold. Thermal Pain Reaction Time: Mice were habituated on a 55°C hot plate for 30 min. The time taken for paw lifting or licking was recorded as the thermal pain reaction time. Motor Nerve Conduction Velocity (MNCV): Under anaesthesia, the sciatic nerve was exposed. Stimulating electrodes were placed at the nerve bifurcation and recording electrodes in the gastrocnemius muscle. Latency of muscle action potential was recorded, and MNCV was calculated as: MNCV (m/s) = Distance between electrodes (mm)/Latency (ms) Sensory Nerve Conduction Velocity (SNCV): Stimulating and recording electrodes were placed at the tibial nerve (ankle) and sciatic nerve (caudal end), respectively. Latency of sensory nerve potential was recorded, and SNCV was calculated as: SNCV (m/s) = Distance between electrodes (mm)/Latency (ms).

#### 2.3.3 Pathological Staining

Mice sciatic nerve tissues were fixed in 4% paraformaldehyde, dehydrated with ethanol, embedded in paraffin, and cut into 5 μm sections. HE, Nissl and TUNEL staining were performed according to previous studies [19, 22]. The Nissl bodies and the proportion of TUNEL‐positive cells were quantified using ImageJ.

For RSC96 cells, TUNEL staining was also conducted, and the proportion of TUNEL positive cells was quantified using ImageJ.

#### 2.3.4 Western Blot

Total proteins of sciatic nerve tissues and RSC96 cells were isolated. Then, the protein concentration was quantified using the BCA method. The expression of target proteins was conducted using western blot as described previously [20]. Image J was used to quantify the grey values of bands in western blot.

#### 2.3.5 LDH And NGF Detection

The levels of LDH and NGF in total protein of sciatic nerve tissues and RSC96 cell supernatants were tested based on the instructions of related commercial kits.

#### 2.3.6 Statistical Analysis

Statistical analysis was performed using SPSS Pro, and all data were expressed as mean ± SD. We applied the Shapiro–Wilk test to evaluate the normality of the data distribution. To assess the significance of differences between groups, the student's unpaired t‐test was utilised, as well as one‐way or two‐way ANOVA, with Tukey's and Bonferroni's post hoc tests for multiple comparisons. If the data did not follow a normal distribution, the rank‐sum test was employed for analysis. For statistical significance, we set a threshold of a *p*‐value of less than 0.05 (*p* < 0.05).

## 3 Results

### 3.1 PMS Intervention Reduced HG‐Induced RSC96 Cell Damage

**FIGURE 1:**
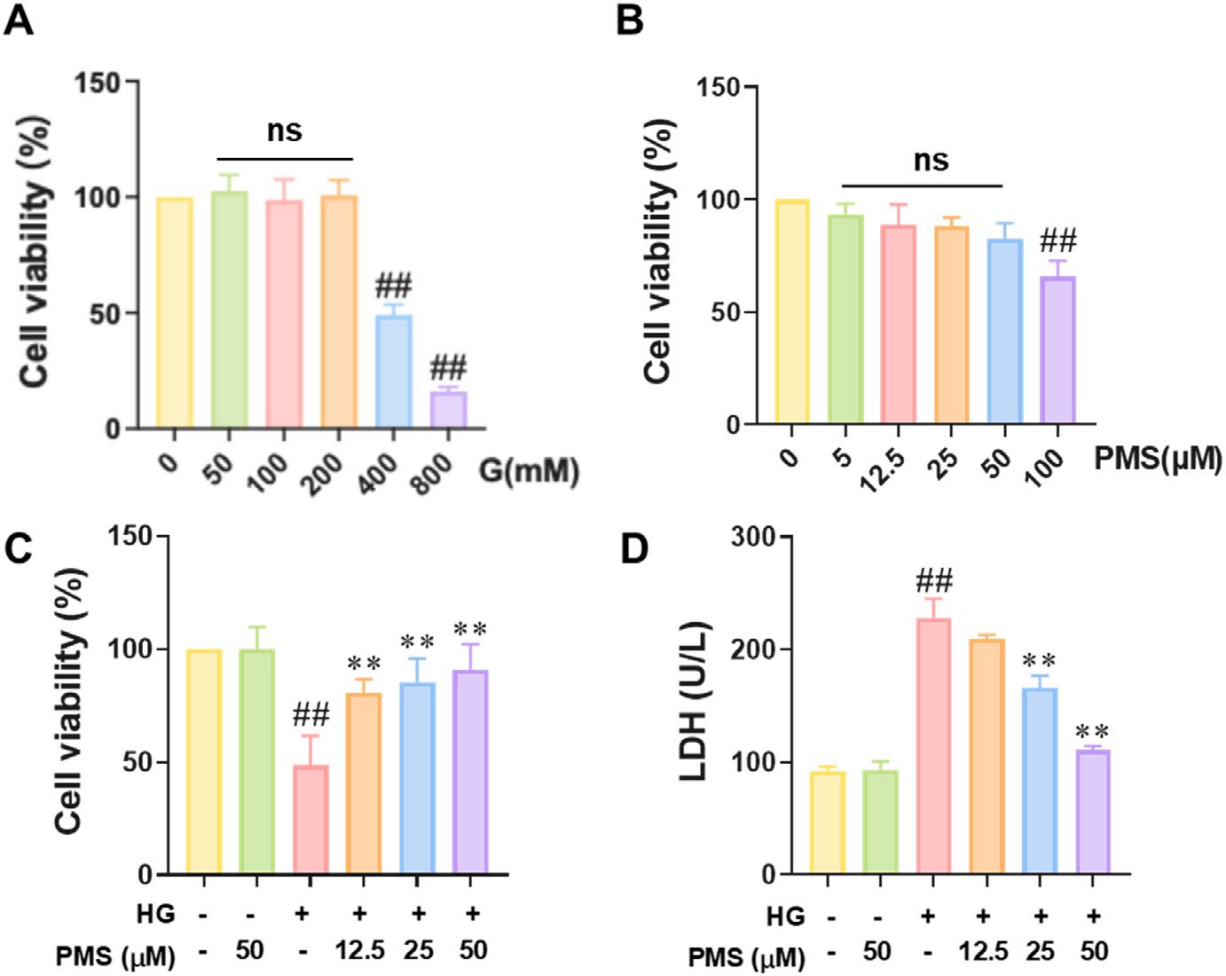
PMS intervention reduced HG‐induced RSC96 cell damage. (A) The MTT assay results of RSC96 cells subjected to different glucose concentration gradients revealed that 400 mM glucose reduced the survival rate of RSC96 cells by approximately 50%, making it suitable as a high glucose condition to induce RSC96 cell damage for establishing an in vitro model of DPN. (B) The MTT assay results of RSC96 cells treated with various PMS concentration gradients indicated that PMS interventions at or below 50 μM had no significant impact on RSC96 cell viability. Therefore, 12.5, 25 and 50 μM PMS were selected as the intervention doses. (C) PMS enhanced the viability of HG‐induced RSC96 cells in a concentration‐dependent manner. (D) PMS reduced LDH activity in a concentration‐dependent manner. Cell groups were divided as follows: Control group, Control + H‐PMS (50 μM) group, HG group (400 mM), HG + L‐PMS (12.5 μM) group, HG + M‐PMS (25 μM) group, and HG + H‐PMS group. Data are presented as mean ± SD. *n* = 6 for A and B, *n* = 10 for C. ##*p* < 0.01 versus Control group; **p < 0.01 versus HG group; ns: No significant (*p* > 0.05). One‐way or two‐way ANOVA followed by post hoc analysis with Bonferroni test for comparison between more groups.

Firstly, we exposed RSC96 cells to different concentrations of glucose to screen for the appropriate glucose concentration for establishing an in vitro model of DPN. The MTT results showed that 400 mM glucose reduced the viability of RSC96 cells by approximately 50%, while 800 mM glucose intervention reduced the viability of RSC96 cells to nearly 20% (Figure 1A). Therefore, we selected 400 mM glucose as the high‐glucose condition to induce RSC96 cell damage for establishing the in vitro model of DPN. Next, we treated RSC96 cells with different concentrations of PMS to determine the optimal PMS intervention concentrations. The MTT results indicated that RSC96 cell viability did not significantly change after treatment with 50 μM or lower concentrations of PMS, while 100 μM PMS significantly reduced RSC96 cell viability (Figure 1B). Therefore, we chose 12.5, 25 and 50 μM PMS to intervene in HG‐induced RSC96 cells to evaluate the effects of PMS on the in vitro model of DPN. The MTT results revealed that compared with the Control group, the cell viability in the HG group was significantly reduced, while PMS enhanced cell viability in a concentration‐dependent manner (Figure 1C). The LDH assay results showed that compared with the Control group, LDH activity in the supernatant of cells in the HG group was significantly enhanced, while PMS reduced LDH activity in a concentration‐dependent manner (Figure 1D). Therefore, in subsequent experiments, we selected 50 μM PMS for exploring the relevant mechanisms.

### 3.2 Transcriptomic Analysis of PMS Intervention in HG‐Induced RSC96 Cells

**FIGURE 2:**
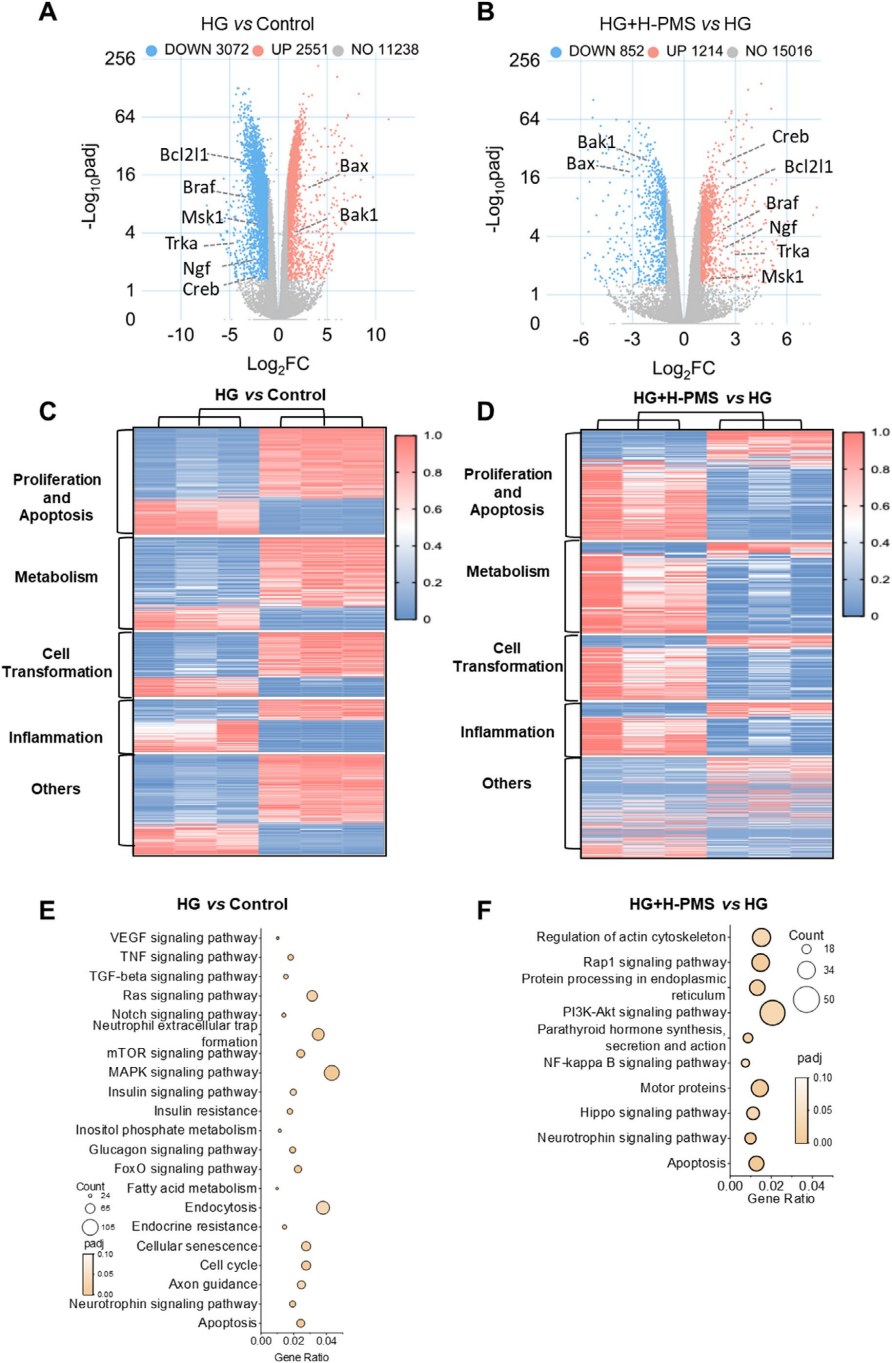
Transcriptomic analysis of PMS intervention in HG‐induced RSC96 cells. We divided the cells into Control, HG, and HG + H‐PMS groups and performed transcriptomic analysis. Differential expression genes (DEGs) between HG versus Control and H‐PMS versus HG were screened using the criteria of |Log_2_(FoldChange)| ≥ 1 and *p*adj ≤ 0.05. (A, B) The DEGs were visualised using volcano plots. (C, D) The relative expression of DEGs was normalised using Min‐Max normalisation method and was visualised by heatmaps. Red represents higher expression genes and blue represents lower expression genes. (E) DEGs in the HG versus Control comparison were significantly enriched in apoptosis, neurotrophin signalling pathway, notch signalling pathway and cell cycle. (F) DEGs in the H‐PMS versus HG comparison exhibited rescue‐associated enrichment in apoptosis, neurotrophin signalling pathway, hippo signalling pathway, etc. Notably, Apoptosis and Neurotrophin signalling pathway were common pathways found in both comparisons. *n* = 3 per group.

Next, we performed transcriptome analysis on cells from the Control group, HG group and HG + H‐PMS group. We screened for differentially expressed genes (DEGs) between HG versus Control and H‐PMS vs. HG based on the criteria of |Log_2_(FoldChange)| ≥ 1 and *p*adj ≤ 0.05 (Figure 2A,B), and presented the expression profiles of these DEGs using heatmaps (Figure 2C,D). Subsequently, we performed KEGG pathway enrichment analysis on these DEGs, revealing that the DEGs from HG versus Control were mainly enriched in apoptosis, neurotrophin signalling pathway, notch signalling pathway, cell cycle, etc. (Figure 2E), while those from H‐PMS versus HG were primarily enriched in apoptosis, neurotrophin signalling pathway, hippo signalling pathway, etc. (Figure 2F). Among these pathways, Apoptosis and Neurotrophin signalling pathway were common pathways, indicating that PMS may exert its protective effects on DPN primarily through these two pathways. Therefore, we proceeded to evaluate the specific impacts of PMS on these two pathways.

### 3.3 PMS Intervention Inhibits HG‐Induced Apoptosis in RSC96 Cells

**FIGURE 3:**
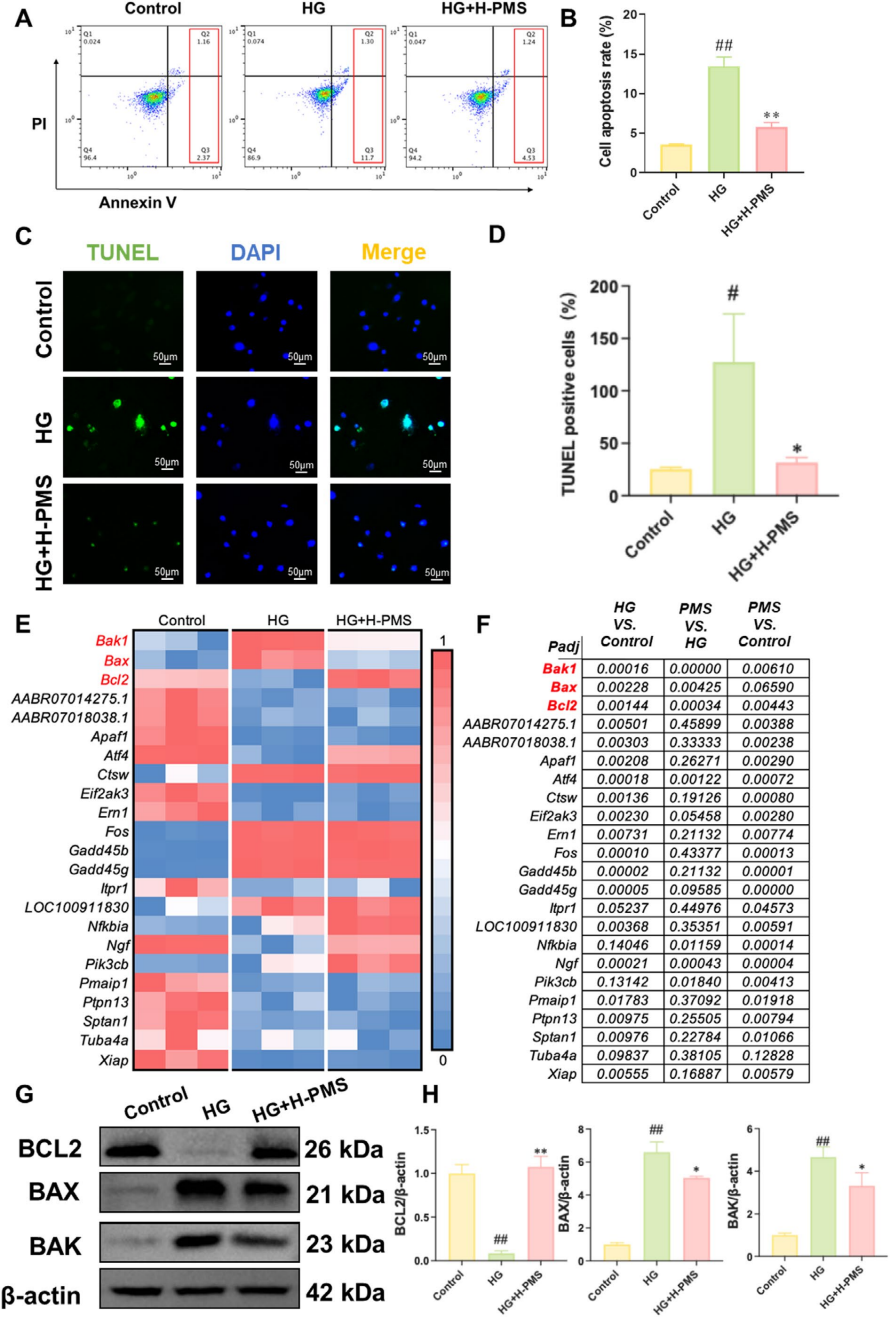
PMS intervention inhibits HG‐induced apoptosis in RSC96 cells. (A, B) Flow cytometry results indicated that PMS intervention significantly reduced the apoptosis rate of HG‐induced RSC96 cells. (C, D) TUNEL staining revealed that PMS intervention significantly reduced the TUNEL‐positive area in HG‐induced RSC96 cells. (E) The relative gene expression related to the Apoptosis pathway was normalised using Min‐Max normalisation method. Red represents higher expression genes and blue represents lower expression genes. The results showed that *Bcl2*, *Bax* and *Bak* were shared DEGs among the Control, HG and HG + H‐PMS groups. PMS intervention significantly upregulated *Bcl2* expression and reduced *Bax* and *Bak* expression. (F) The *p*‐values of differentially expressed genes related to the apoptosis pathway among the Control, HG and HG + H‐PMS groups. (G, H) Western blot analysis demonstrated that PMS intervention significantly upregulated BCL2 protein expression and reduced BAX and BAK protein expression. Cell groups included Control, HG, and HG + H‐PMS. Data are presented as mean ± SD. *n* = 3 per group. #*p* < 0.05, ##*p* < 0.01 versus Control group; *p < 0.05, **p < 0.01 versus HG group. One‐way or two‐way ANOVA followed by post hoc analysis with Bonferroni test for comparison between more groups.

We first evaluated the effect of PMS on HG‐induced apoptosis in RSC96 cells. Flow cytometry results showed that the apoptosis rate of RSC96 cells significantly enhanced after HG intervention, while PMS intervention significantly reduced the apoptosis rate of RSC96 cells (Figure 3A,B). TUNEL staining results revealed that HG intervention enhanced the proportion of TUNEL‐positive cells in RSC96 cells, whereas PMS intervention reversed this effect (Figure 3C,D). Furthermore, we examined the impact of PMS on apoptosis and related genes under normoglycemic conditions (the methods are detailed in the Supporting Information, and the primers are listed in Table S1). The results demonstrated that PMS alone did not significantly alter the viability or apoptosis rate of RSC96 cells, confirming that the protective effects of PMS are strictly dependent on HG‐induced stress (Figure S4A,B). We presented the expression profiles of genes related to the Apoptosis pathway in the Control group, HG group, and HG + H‐PMS group using a heatmap. The results showed that *Bcl2*, *Bax* and *Bak* were shared DEGs among the three groups, and PMS intervention significantly upregulated *Bcl2* expression while downregulating *Bax* and *Bak* expression (Figure 3E,F). Therefore, we further assessed the effects of PMS on the protein expression of BCL2, BAX and BAK using Western blot. The results indicated that compared with the Control group, BCL2 protein expression was significantly reduced, while BAX and BAK protein expressions were significantly upregulated in the HG group. In contrast, PMS intervention significantly upregulated BCL2 protein expression and reduced BAX and BAK protein expression (Figure 3G,H). Additionally, no significant alterations in the gene expression levels of these proteins were observed in normal RSC96 cells subjected to PMS intervention (Figure S4C).

### 3.4 PMS Intervention Activates the NGF/TrkA Pathway in HG‐Induced RSC96 Cells

**FIGURE 4:**
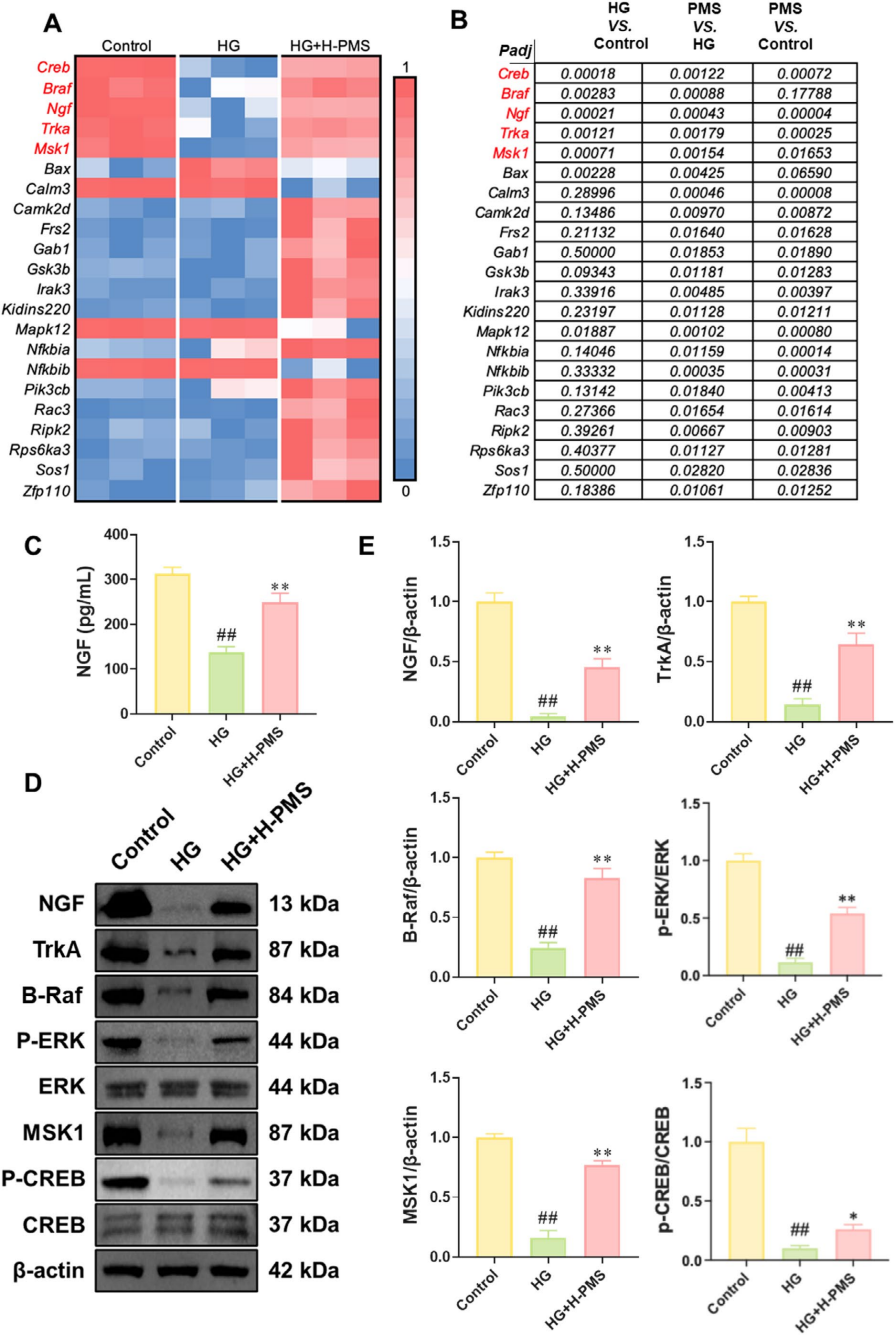
PMS intervention activates the NGF/TrkA pathway in HG‐induced RSC96 cells. (A) The DEGs related to the Neurotrophin pathway were visualised using volcano plots. (C, D) The relative expression of DEGs was normalised using Min‐Max normalisation method and was visualised by heatmaps. Red represents higher expression genes and blue represents lower expression genes. *Ngf, Trka, Braf, Msk1*, and *Creb* were shared DEGs among the Control, HG, and HG + H‐PMS groups, and PMS intervention significantly upregulated the expression of these genes. (B) The *p*‐values of differentially expressed genes related to the Neurotrophin signalling pathway among the Control, HG, and HG + H‐PMS groups. (C) PMS intervention enhanced the level of NGF in the supernatant of HG‐induced RSC96 cells. (D, E) Western blot results showed that the expression of NGF, TrkA, B‐Raf, P‐ERK/ERK, MSK1 and P‐CREB/CREB was significantly upregulated after PMS intervention. Cell groups included Control, HG, and HG + H‐PMS. Data are presented as mean ± SD. *n* = 3 per group. ##*p* < 0.01 versus Control group; *p < 0.05, **p < 0.01 versus HG group. One‐way or two‐way ANOVA followed by post hoc analysis with Bonferroni test for comparison between more groups.

Next, we evaluated the impact of PMS on the Neurotrophin signalling pathway. The heatmap of gene expression revealed that *Ngf*, *Trka*, *Braf*, *Msk1* and *Creb* were shared differentially expressed genes (DEGs) among the Control, HG and HG + H‐PMS groups, and PMS intervention significantly upregulated the expression of these genes (Figure 4A,B). Subsequently, we measured the NGF levels in the cell supernatants of each group, and the results showed that HG intervention significantly decreased the NGF level, whereas after PMS intervention, the NGF level in the HG‐induced RSC96 cell supernatants enhanced significantly (Figure 4C). Furthermore, we used Western blot to detect the expression of NGF, TrkA, B‐Raf, P‐ERK/ERK, MSK1 and P‐CREB/CREB to further validate the effect of PMS on the NGF/TrkA pathway within the Neurotrophin signalling pathway. The results indicated that compared to the Control group, the levels of these proteins were significantly reduced in the HG group, whereas PMS intervention reversed this decrease in protein expression (Figure 4D,E). Additionally, no significant alterations in the gene expression levels of these proteins were observed in normal RSC96 cells subjected to PMS intervention (Figure S4C).

### 3.5 Inhibiting the NGF/TrkA Pathway Attenuates the Inhibitory Effect of PMS on HG‐Induced Apoptosis in RSC96 Cells

**FIGURE 5:**
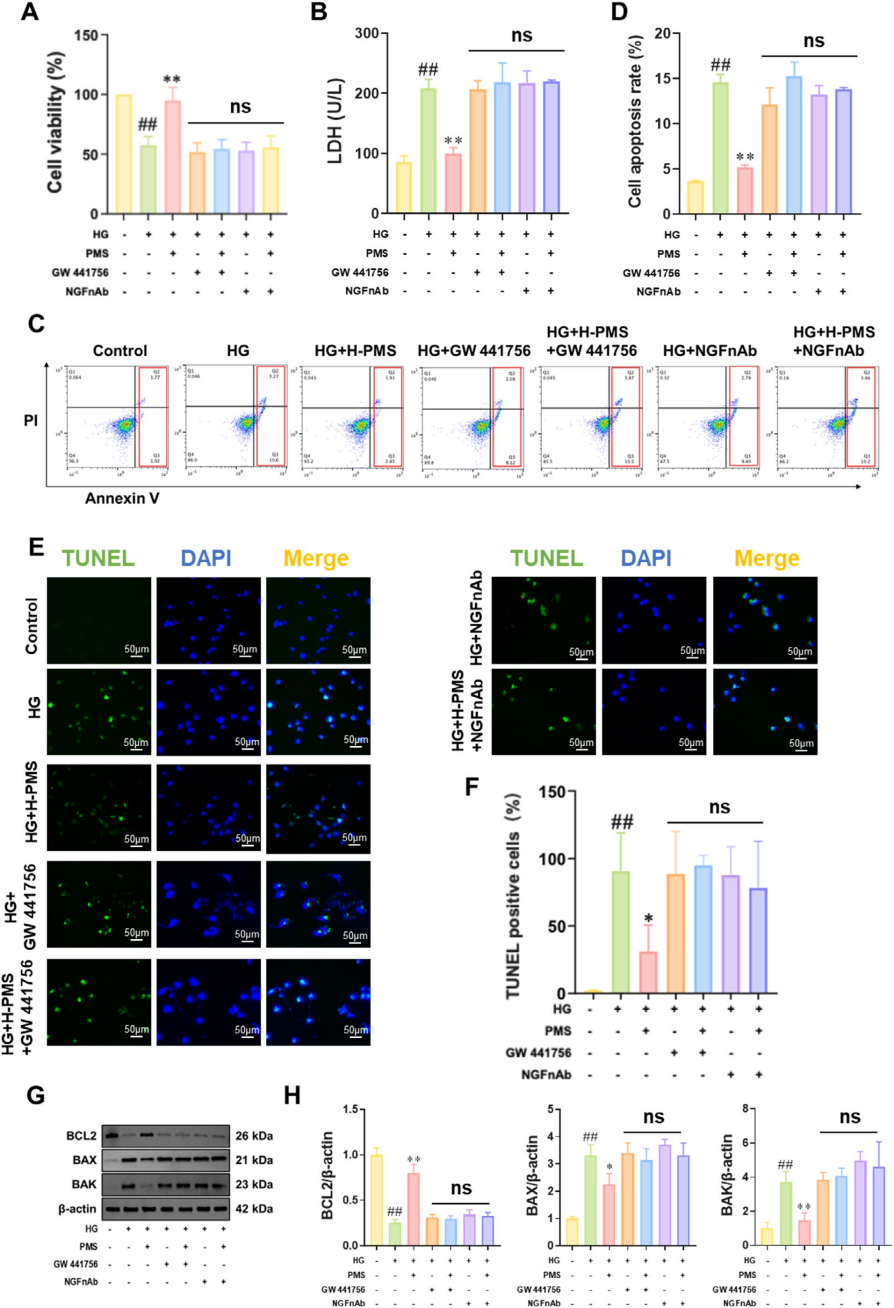
Inhibiting the NGF/TrkA pathway attenuates the inhibitory effect of PMS on HG‐induced apoptosis in RSC96 cells. To validate the role of PMS in activating the NGF/TrkA pathway and inhibiting apoptosis, we performed experiments by inhibiting NGF and TrkA separately. (A) MTT results showed that PMS intervention significantly improved the survival rate of HG‐induced RSC96 cells, while this effect was diminished or even abolished when combined with GW 441756 or NGFnAb. (B) LDH assay results indicated that PMS intervention enhanced LDH activity, but this enhancement was attenuated by GW 441756 or NGFnAb intervention. (C, D) Flow cytometry results revealed that GW 441756 or NGFnAb intervention weakened the ability of PMS to reduce apoptosis. (E, F) TUNEL staining showed that GW 441756 or NGFnAb intervention attenuated the reduction in TUNEL‐positive area by PMS. (G, H) Western blot results demonstrated that after GW 441756 or NGFnAb intervention, the upregulation of BCL2 by PMS and the downregulation of BAX and BAK by PMS were abolished. Data are presented as mean ± SD. *n* = 6 for (A); *n* = 10 for (B, C); *n* = 3 for (D, E). ##*p* < 0.01 versus Control group; *p < 0.05, **p < 0.01 versus HG group; ns: No significant (*p* > 0.05). One‐way or two‐way ANOVA followed by post hoc analysis with Tukey's test for comparison between more groups.

To further validate whether PMS inhibits HG‐induced apoptosis in RSC96 cells by promoting the NGF/TrkA pathway, RSC96 cells were treated with TrkA inhibitor (GW 441756, 1 μM) and NGFnAb (10 μg/mL) for 24 h [26], respectively. MTT results showed that PMS intervention significantly enhanced the survival rate of HG‐induced RSC96 cells, while this promoting effect on cell viability was weakened or even abolished in the presence of GW 441756 or NGFnAb (Figure 5A). LDH assay results indicated that compared to the Control group, LDH activity was significantly decreased in the HG group, and PMS intervention enhanced LDH activity, whereas GW 441756 or NGFnAb intervention attenuated the enhancing effect of PMS on LDH activity (Figure 5B). Both flow cytometry and TUNEL assays demonstrated that GW 441756 or NGFnAb intervention weakened the effect of PMS in reducing the apoptosis rate and the proportion of TUNEL‐positive cells (Figure 5C–F). Western blot results revealed that after GW 441756 or NGFnAb intervention, the upregulation of BCL2 by PMS and the downregulation of BAX and BAK by PMS were abolished (Figure 5G,H).

### 3.6 PMS Activates the NGF/TrkA Pathway in DPN Mice, Inhibits Neuronal Apoptosis, and Exerts an Ameliorating Effect on DPN Behaviour and Neuroprotection

**FIGURE 6:**
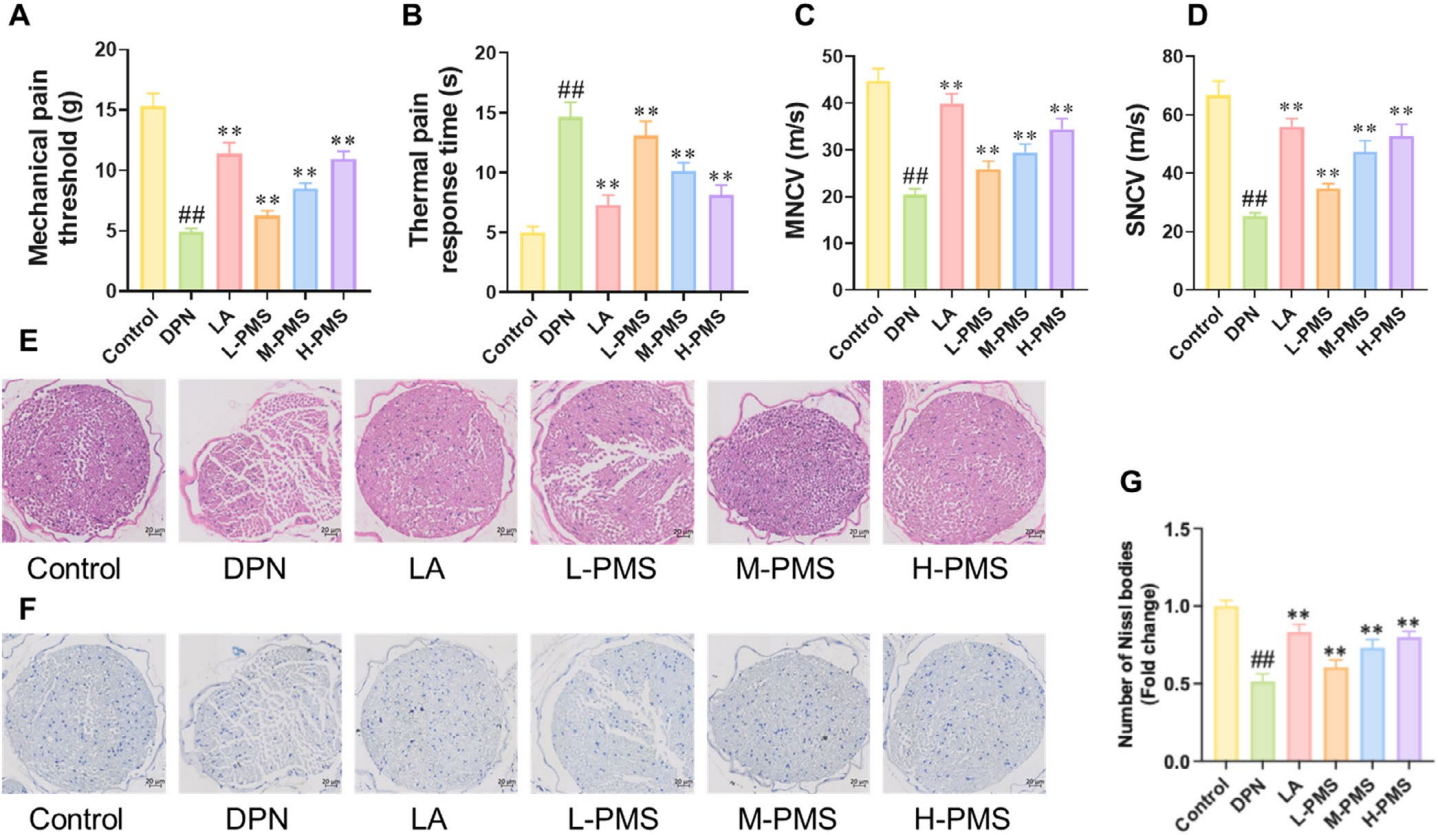
PMS exerts an ameliorating effect on DPN behaviour and neuroprotection. We further established a diabetic peripheral neuropathy (DPN) mouse model via STZ injection (150 mg/kg) and administered PMS orally at doses of 25, 50 and 100 mg/kg/day, to validate the effect of PMS on improving DPN through in vivo experiments. (A–D) Behavioural tests showed that PMS dose‐dependently improved the mechanical pain threshold (A), thermal pain response time (B), motor nerve conduction velocity (C) and sensory nerve conduction velocity (D) in DPN mice. (E) HE staining results indicated that PMS intervention effectively ameliorated pathological damages such as loose and disorganised sciatic nerve fibre structures and disturbed Schwann cell distribution. (F, G) Nissl staining results revealed that PMS intervention dose‐dependently enhanced the number of Nissl bodies. Data are presented as mean ± SD. *n* = 6 for (A); *n* = 10 per group. ##*p* < 0.01 versus Control group; **p < 0.01 versus HG group. One‐way or two‐way ANOVA followed by post hoc analysis with Tukey's test for comparison between more groups. MNCV, motor nerve conduction velocity; SNCV, sensory nerve conduction velocity.

**FIGURE 7:**
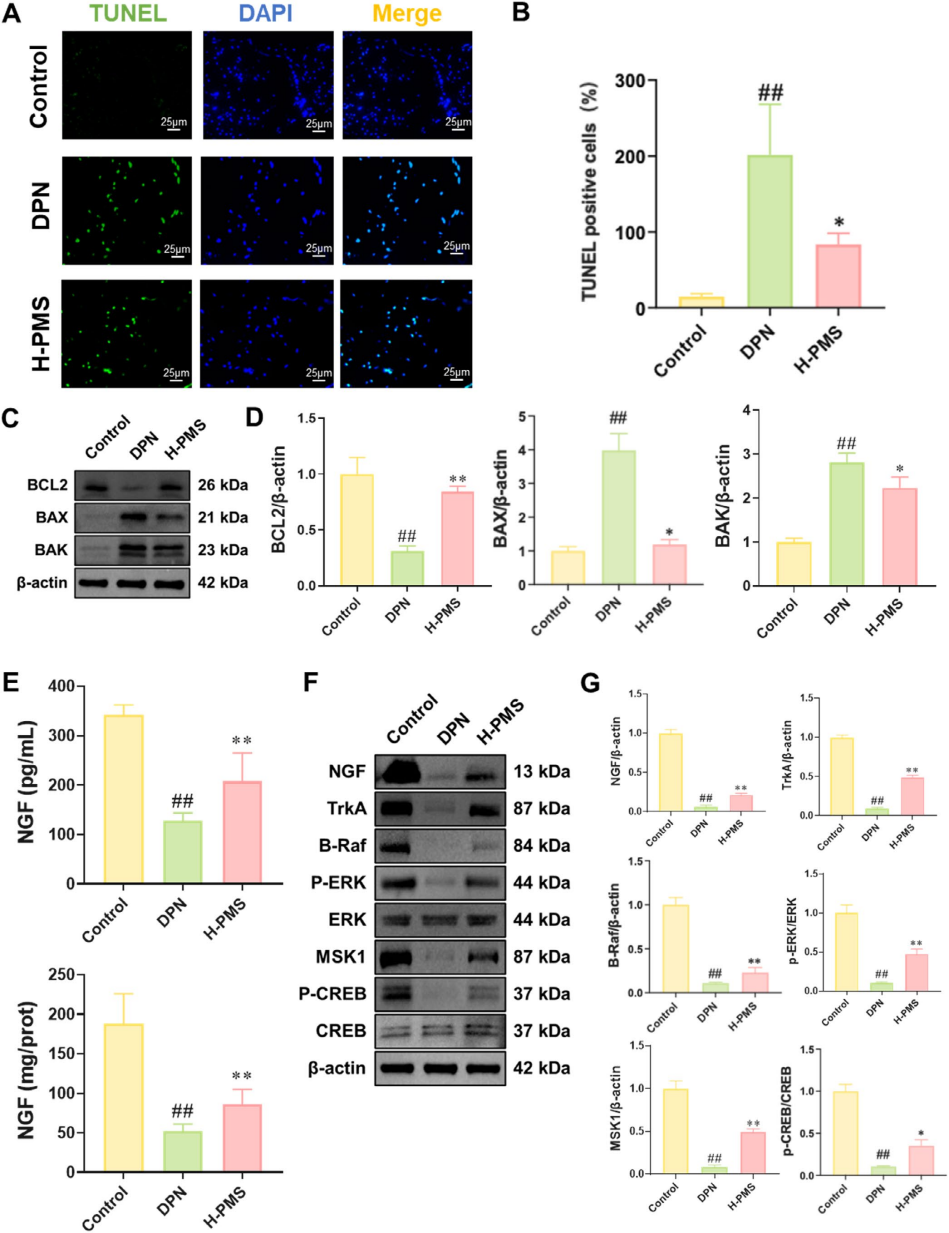
PMS activates the NGF/TrkA pathway in DPN mice, thereby inhibiting neuronal apoptosis. Based on the validation of PMS's effect on improving DPN in mice, we further investigated the mechanism of PMS in vivo. (A, B) TUNEL staining results showed that PMS intervention significantly reduced TUNEL‐positive expression in the sciatic nerve of DPN mice. (C, D) Western blot results indicated that PMS intervention reversed the decreased BCL2 protein expression and enhanced BAX and BAK protein expression in the sciatic nerve of DPN mice. (E) NGF assay results demonstrated that PMS intervention significantly upregulated NGF levels in the serum and sciatic nerve tissue of DPN mice. (F, G) Western blot results showed that PMS intervention significantly upregulated the expression of NGF, TrkA, B‐Raf, P‐ERK/ERK, MSK1 and P‐CREB/CREB in the sciatic nerve of DPN mice. Data are presented as mean ± SD. *n* = 3 for (A, B and D); *n* = 10 for (C). ##*p* < 0.01 versus Control group; *p < 0.05, **p < 0.01 versus HG group. One‐way or two‐way ANOVA followed by post hoc analysis with Tukey's test for comparison between more groups.

In vivo study was conducted to verify the therapeutic effects and the mechanisms of PMS. Briefly, DPN was induced in mice through STZ injection. Then, DPN mice received oral treatment of 25, 50 and 100 mg/kg of PMS. LA was used as the positive control. Behavioural tests revealed that compared to the Control group, DPN mice exhibited significantly decreased mechanical pain threshold, enhanced thermal pain reaction time, and reduced motor and sensory nerve conduction velocities. PMS administration improved these behavioural outcomes (Figure 6A–D). HE staining showed that the sciatic nerve fibres of Control mice were neatly structured, tightly arranged, and uniformly stained for myelin sheaths, with scattered Schwann cells observed between nerve fibres. In contrast, DPN mice exhibited loose and disorganised sciatic nerve fibres, with uneven myelin sheath staining and patchy desquamation. PMS intervention effectively ameliorated the pathological damage to the sciatic nerve (Figure 6E). Nissl staining demonstrated a significant reduction in the number of Nissl bodies in the sciatic nerve of DPN mice, which was dose‐dependently enhanced by PMS treatment (Figure 6F,G). Furthermore, when using LA as a positive drug, we found that high‐dose PMS intervention produced similar effects to LA in treating DPN. Therefore, we selected high‐dose PMS for subsequent mechanistic validation experiments. TUNEL staining revealed that compared to the Control group, the area of TUNEL‐positive regions in the sciatic nerve of DPN mice was significantly enhanced, while PMS intervention significantly reduced TUNEL‐positive expression (Figure 7A,B). Western blot analysis showed that compared to the Control group, BCL2 protein expression was significantly decreased, while BAX and BAK protein expressions were markedly enhanced in the sciatic nerve of DPN mice. PMS intervention reversed these changes (Figure 7C,D). NGF measurements indicated that both serum and sciatic nerve tissue NGF levels were significantly reduced in DPN mice, whereas PMS treatment significantly upregulated NGF levels (Figure 7E). Western blot analysis further confirmed that the expression of NGF, TrkA, B‐Raf, P‐ERK/ERK, MSK1 and P‐CREB/CREB was significantly decreased in the sciatic nerve of DPN mice compared to the Control group, and PMS intervention significantly upregulated the expression of these proteins (Figure 7F,G).

## 4 Discussion

The degenerative changes in nerve fibres, damage to nerve cells, and functional impairments caused by DPN have severely impacted patients' quality of life [27]. Hyperglycemia is a critical factor in the onset and progression of DPN [28]. Under hyperglycemic conditions, irreversible non‐enzymatic reactions occur between glucose and proteins, lipids, and nucleic acids, resulting in the formation of end products that induce apoptosis in endothelial cells, smooth muscle cells, and nerve cells through multiple pathways [29]. Studies have shown a significant positive correlation between hyperglycemic states in diabetic patients and LDH activity [30]. Another study revealed that under HG intervention, the apoptosis rate of neurons increases dramatically [31], with LDH serving as an important marker for evaluating cellular apoptosis [32]. The RSC96 Schwann cell line is a recognised in vitro model for studying DPN. The in vitro model of DPN was established by exposing RSC96 Schwann cells to high glucose, which induced cellular apoptosis [33, 34]. Our results demonstrate that HG induces apoptosis in RSC96 cells, evident by significantly enhanced LDH activity, a marked increase in apoptosis rates, and a notable expansion in the proportion of TUNEL‐positive cells. This indicates that HG‐induced RSC96 cell damage has successfully established an in vitro model of DPN. Notably, PMS intervention significantly improves RSC96 cell apoptosis, highlighting its tremendous potential in DPN treatment. Furthermore, impaired pancreatic islet function leading to insulin deficiency is another crucial factor exacerbating DPN. Insulin deficiency further promotes hyperglycemia in the body, rapidly intensifying the damaging effects of high glucose on nerve tissues [32]. In our in vivo experiments, we induced islet damage in mice through intraperitoneal injection of STZ to establish a mouse model of DPN, a widely adopted modelling method [22]. While LA has been extensively used as a positive drug in DPN animal models [35], our experimental results reveal that high doses of PMS exhibit comparable efficacy to LA. Our results suggest the neuroprotective potential of PMS. Consistently, the neuroprotective effects of PMS have also been reported in an acute spinal cord injury [36] model. These findings collectively highlight the unique advantages of PMS in treating Neuronal injury diseases. Importantly, although natural products such as quercetin have been shown to exert neuroprotective potential, their clinical applications are hampered by pharmacokinetic limitations, including poor water solubility, low oral bioavailability, and rapid first‐pass metabolism [37, 38]. In contrast, PMS may demonstrate superior bioavailability due to its favourable aqueous solubility and potential bioactive metabolites [18]. Subsequent research should compare the structure–activity relationships, pathway specificity, and clinical translation potential of different natural products to advance the development of novel neuroprotective agents. In addition, a positive control group was missed in in vitro studies. Our future study will use relative positive control drugs to further validate the neuroprotective effect in vitro.

Subsequently, our transcriptomic analysis of HG‐induced RSC96 cells under PMS intervention identified Apoptosis and Neurotrophin signalling pathway as the key pathways mediating PMS's therapeutic effects on DPN. In validating PMS's inhibition of Apoptosis, we observed that PMS intervention significantly upregulated BCL2 expression while downregulating BAX and BAK expression. BCL2, an anti‐apoptotic protein located on the outer mitochondrial membrane, is a crucial factor involved in the regulation of cellular apoptosis. BAX belongs to the BCL2 family and functions as pro‐apoptotic proteins on the outer mitochondrial membrane. BCL2 can bind to BAX, thereby exerting an anti‐apoptotic effect. Under physiological conditions, BAX remains inactive, but when cells are stimulated by high glucose or other factors, BAX forms homodimers with itself, translocates from the cytoplasm to the outer mitochondrial membrane, and initiates apoptosis. BAK shares similar functions with BAX, also belonging to the BCL2 family and promoting apoptosis on the outer mitochondrial membrane [18]. However, like BAX, BAK can lose its pro‐apoptotic activity when bound by BCL2. Typically, the expression levels of BCL2, BAX and BAK remain relatively stable within cells. However, when BAX or BAK is overexpressed, they counteract the inhibitory effects of apoptosis and participate in promoting apoptosis. Conversely, when BCL2 is overexpressed, it halts the cascade amplification of apoptosis in mitochondria, reverses morphological changes in cells, and inhibits apoptosis‐inducing effects to participate in apoptosis suppression [39, 40, 41]. Studies have found that Schwann cells, when stimulated by high glucose, undergo changes in the redistribution of BAX and conformation of BCL2, BAX and BAK within the cells, triggering a series of cascade reactions that promote apoptosis [42]. Inhibitors of BAX or BAK can effectively downregulate the expression of BAX or BAK or block their redistribution, thereby exerting an anti‐apoptotic effect [43]. Our findings support PMS's inhibitory effect on BAX and BAK, suggesting that PMS may improve DPN through its anti‐apoptotic mechanism. Live‐cell imaging technique can be used in future studies to evaluate the changes in BAX redistribution following PMS treatment. Furthermore, the effects of PMS on conformational changes in BCL2, BAX and BAK can be studied by co‐immunoprecipitation.

In validating PMS's promotion of the Neurotrophin signalling pathway, we discovered that PMS intervention significantly upregulated the expression of NGF, TrkA, B‐Raf, P‐ERK/ERK, MSK1 and P‐CREB/CREB. This suggests that PMS can regulate the NGF/TrkA pathway. NGF, an essential neurotrophic factor, plays a pivotal role in the survival, growth, and differentiation of nerve cells [44]. During the progression of DPN, NGF deficiency‐induced impairments in neurotrophic signalling exacerbate high glucose‐induced neurodegenerative processes [45]. NGF promotes myelin formation, activates the self‐cleansing function of Schwann cells, and accelerates peripheral nerve regeneration [46]. The expression and function of NGF may be affected by high glucose environments, thereby influencing the survival and function of nerve cells [47]. TrkA, the specific binding receptor for NGF, activates various downstream pathways upon specific binding with NGF, exerting anti‐apoptotic and neuroprotective effects [48]. Deficiency of neurotrophic factors can lead to neurological dysfunction. Research has found that NGF‐containing agents, such as heparin‐poloxamer hydrogels, can promote peripheral nerve regeneration in diabetic rats, which may be related to the activation of signalling pathways such as B‐Raf/ERK/MSK1 [49]. B‐Raf, a member of the growth signal transduction protein kinase family, plays a role in regulating the ERK/MSK1 signalling pathway, thereby influencing cell division, differentiation, and apoptosis [50]. B‐Raf is preferentially expressed in cells of neuronal origin, including neural stem cells, neural progenitor cells, and differentiated neurons. Furthermore, conditional ablation of B‐Raf in neural progenitor cells has been shown to result in severe myelination disorders, impaired oligodendrocyte differentiation, and diminished ERK (Extracellular signal‐regulated kinase) activation in the brain [51]. ERK, a widely expressed protein kinase intracellular signalling molecule, participates in regulating meiosis, mitosis, and post‐mitotic functions in differentiated cells. After B‐Raf activates ERK, ERK undergoes phosphorylation and enters the nucleus to promote MSK1 activation, which in turn promotes CREB phosphorylation. On one hand, CREB, as the terminal transcription factor of the Neurotrophin signalling pathway, is widely and fully expressed in nerve cells. Phosphorylated CREB regulates neuronal growth, differentiation, proliferation, synaptic plasticity, neurogenesis, neuronal maturation, spatial memory, long‐term memory formation, and neuronal survival [52]. Research has shown that CREB can serve as a key target for neurodegenerative complications, particularly Alzheimer's disease [52]. Additionally, studies have demonstrated a significant positive correlation between CREB phosphorylation and Schwann cell proliferation [53]. On the other hand, phosphorylated CREB can promote BCL2 expression, inhibiting apoptosis through the formation of BCL2/BAX or BCL2/BAK dimers [54]. Furthermore, NGF neutralising antibody and TrkA inhibitor (GW441756) abolished the anti‐apoptotic effect of PMS. These data indicate that PMS may inhibit cell apoptosis and improve DPN pathological damage by activating the NGF/TrkA pathway. Genetic KO/KD methods (CRISPR or RNAi) can also be used in future studies to further verify this finding. Moreover, in vivo results further support this conclusion.

Furthermore, transcriptomics data show a stronger rescue effect compared to the western blot data in our study. As transcriptomic data reflect the profiles of gene expression at specific time points, the differences between transcriptomic and western blot data may be related to the discrepancy in gene and protein expression at such time points. These may be caused by epigenetic modifications, post‐transcriptional regulation, and post‐translational modifications [55]. Further studies can also focus on the epigenetic, post‐transcriptional and post‐translational regulatory mechanisms of PMS. Notably, we primarily focused on the effects of PMS on DPN models in this study; high‐dose PMS significantly ameliorated DPN pathological changes without inducing adverse outcomes. While the impact of PMS on healthy mice was not investigated in the present study, previous research indicates that PMS treatment does not exhibit any adverse effects on healthy mice [56]. Future studies incorporating PMS intervention in healthy mice would facilitate the evaluation of its safety profile and the specificity of its therapeutic mechanisms.

The anti‐inflammatory effects of PMS have also been reported by previous studies. Studies have shown that PMS alleviates inflammatory responses by inhibiting the activation of the NF‐κB signalling pathway and reducing the release of pro‐inflammatory cytokines such as TNF‐α and IL‐6. Notably, the inflammatory microenvironment is closely associated with the NGF signalling pathway. Research has confirmed that pro‐inflammatory cytokines like TNF‐α and IL‐6 can significantly upregulate NGF expression by activating the NF‐κB pathway [57], and the overactivation of the NGF/TrkA signalling pathway is closely linked to the pathological progression of various inflammation‐related diseases. In oesophageal squamous cell carcinoma, PMS reduces IL‐6 secretion by targeting the IL‐6/STAT3 pathway [58]. This inhibition of IL‐6 may influence NGF signalling through dual mechanisms: on one hand, IL‐6 itself directly stimulates glial cells to produce NGF; On the other hand, there is cross‐regulation between the IL‐6/STAT3 pathway and TrkA receptor phosphorylation [59]. Thus, PMS‐mediated suppression of IL‐6 may indirectly regulate NGF/TrkA pathway activity. Animal experiments further demonstrate that PMS extracts reduce levels of pro‐inflammatory factors such as IL‐6 and TNF‐α in colitis models [60]. Considering the unique role of NGF in intestinal inflammation, where it not only participates in pain sensitisation but also mediates epithelial barrier repair via the TrkA receptor, we speculate that PMS may indirectly affect the biological effects of the NGF/TrkA signalling pathway by modulating the dynamic balance between pro‐inflammatory and anti‐inflammatory factors.

**FIGURE 8:**
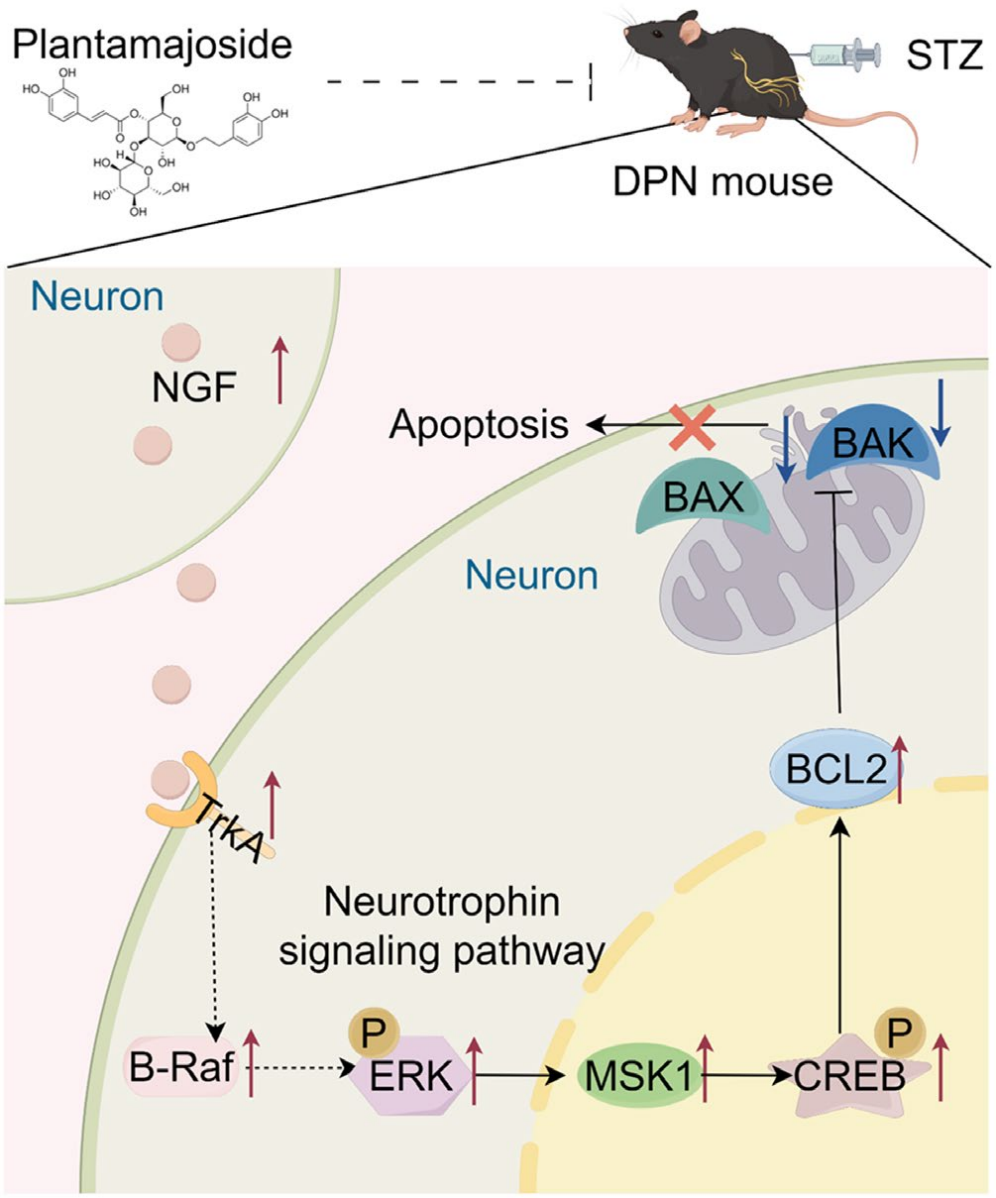
PMS activates the NGF/TrkA pathway, which in turn inhibits cell apoptosis, ultimately leading to improved nerve damage in DPN.

In summary, our research has clearly demonstrated the significant potential of PMS in improving DPN‐related nerve damage both in vivo and in vitro. The underlying mechanism may involve PMS activating the NGF/TrkA pathway, thereby inhibiting cell apoptosis and ultimately promoting the recovery of DPN‐affected nerve function (Figure 8). However, this is merely a preliminary study on the efficacy and mechanism. A more comprehensive picture of the PMS treatment mechanism for DPN requires further experimentation, integrating targeted metabolomics, single‐cell RNA sequencing, target prediction and validation, among other approaches.

## Author Contributions

**Qingshan Hai:** funding acquisition (equal), investigation (equal), writing – original draft (equal). **Yuming Wang:** data curation (equal), investigation (equal), validation (equal). **Hanzhou Li:** investigation (equal), validation (equal), visualization (equal). **Huan Pei:** formal analysis (equal), investigation (equal), validation (equal). **Ning Wang:** investigation (equal). **Xiaoxia Zhang:** visualization (equal). **Mingyao Fan:** validation (equal). **Jiabao Liao:** validation (equal). **Weibo Wen:** conceptualization (equal), supervision (equal). **Jie Zhao:** conceptualization (equal), supervision (equal). **Ling Yang:** conceptualization (equal), supervision (equal). **Huantian Cui:** conceptualization (equal), writing – review and editing (equal).

## Disclosure

All data were generated in‐house, and no paper mill was used. All authors agreed to be accountable for all aspects of the work ensuring integrity and accuracy.

## Conflicts of Interest

The authors declare no conflicts of interest.

## Supporting information


Data S1.

## Data Availability

Data will be made available on request.
