# Supplementary material for: Plantamajoside Promotes NGF/TrkA Pathway to Inhibit Neuronal Apoptosis and Improve Diabetic Peripheral Neuropathy
Source: J Cell Mol Med. 2025 Apr 28;29(8):e70571. doi: 10.1111/jcmm.70571 (PMC12034938; doi:10.1111/jcmm.70571)
Supplement: Supplementary file 1 — Data S1. [file JCMM-29-e70571-s001.docx]

**Supplementary Materials**

**Materials and Methods**

**Reagents**

Lipoic acid (T1395, Empirical formula: C_8_H_14_O_2_S_2_, Purity ≥99%, Constitutional formula was shown in Figure S1), Plantamajoside (PHL83304, Empirical formula: C_29_H_36_O_16_, Purity ≥95%, Constitutional formula was shown in Figure S2), Streptozotocin (V900890, Empirical formula: C_8_H_15_N_3_O_7_, Purity ≥98%, Constitutional formula was shown in Figure S3) were purchased from Merck China (Beijing, China). Annexin V-APC/Pl Apoptosis Kit (AP107) was purchased from MULTI SCIENCES (Hangzhou, China). LDH assay kit (A020-2-2) was purchased from Nanjing Jiancheng Bioengineering Research Institute Co., LTD.(Nanjing, China). ELISA kit for NGF (ml202835) was purchased from Shanghai Enzyme-linked Biotechnology Co., Ltd. (Shanghai, China). GW441756 (54513ES10) was purchased from YEASEN (Shanghai, China). NGFnAb (N8773) was purchased from Merck China (Beijing, China). Primary antibodies: BCL2 (ab194583, 1/1000), BAX (ab32503, 1/5000), were purchased from Abcam (Shanghai, China). BAK (PA5-104224, 1/1000), NGF (PA5-29425, 1/1000), TrkA (MA5-32123, 1/5000), B-Raf (PA5-81931, 1/1000), P-ERK (44-680G, 1/1000), ERK (MA5-15134, 1/1000), MSK1 (PA5-36099, 1/1000), P-CREB (MA5-11192, 1/1000), CREB (MA1-083, 1/500) were purchased from Thermo Fisher Scientific (Shanghai, China). Secondary antibody for goat anti-mouse IgG H&L (ab6785) and goat anti-rabbit IgG H&L (ab207995) were purchased from Abcam (Shanghai, China).

**RT-qPCR**

Total RNA was extracted from RSC96 cell pellets and cells cultured in vitro using TRIzol® (Life Technologies, Inc., USA), according to the manufacturer's instructions; the RNA (1μg) from each group was reverse transcribed by a reverse transcription kit (ReverTra Ace® qPCR RT Kit, Toyobo Inc., Japan) to obtain the corresponding cDNA. qPCR was performed to determine the mRNA expression levels of the target genes. The relative expression of each target mRNA to β-actin was calculated using the 2-^ΔΔ^CT method. Primer sequences can be found in the Table S1.

**Table S1** Primer sequence

| Gene | Sequence (5'->3') | |
| --- | --- | --- |
| *Bak1* | Forward primer | GGTGACCTGCTTTTTGGCTG |
|  | Reverse primer | TTACGGTCAGGATGGGGTCT |
| *Bax* | Forward primer | GAACCATCATGGGCTGGACA |
|  | Reverse primer | GGAGAGGAGGCCTTCCCAG |
| *Bcl2* | Forward primer | GGTGACCTGCTTTTTGGCTG |
|  | Reverse primer | TTACGGTCAGGATGGGGTCT |
| *Creb* | Forward primer | CCAGCAGCTCATGCAACATC |
|  | Reverse primer | GCACTGCCACTCTGTTCTCT |
| *Braf* | Forward primer | TTCCCCAAATTCTCGCCTCC |
|  | Reverse primer | GCTGCAAATTCTCCATATCCCC |
| *Ngf* | Forward primer | ACAGGCAGAACCGTACACAG |
|  | Reverse primer | TGACGAAGGTGTGAGTCGTG |
| *Trka* | Forward primer | GGGCTAGGCAGTCTGATGAC |
|  | Reverse primer | CCTCAAACTCCAAGCGTTGC |
| *Msk1* | Forward primer | AACAGGACACGCAGAGAAGG |
|  | Reverse primer | GGAGAGTAGGTGGGGTCCAT |


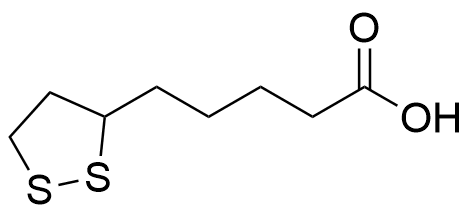


**Figure S1 Constitutional formula of Lipoic acid.**


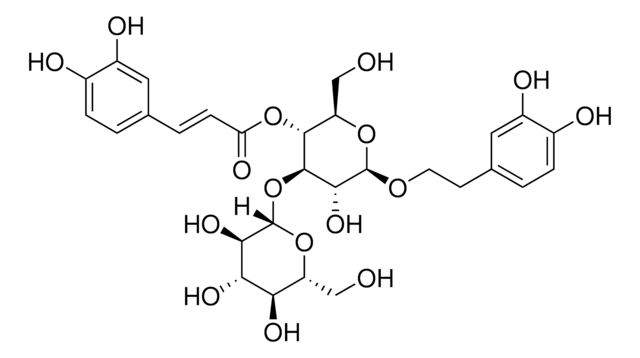


**Figure S2 Constitutional formula of Plantamajoside.**


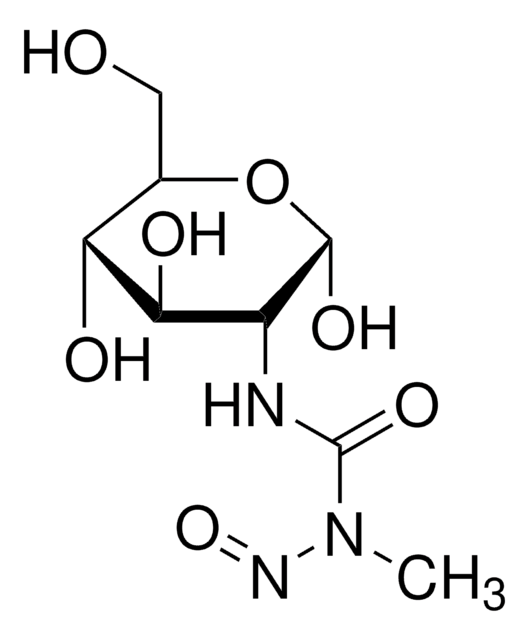


**Figure S3 Constitutional formula of Streptozotocin.**

**
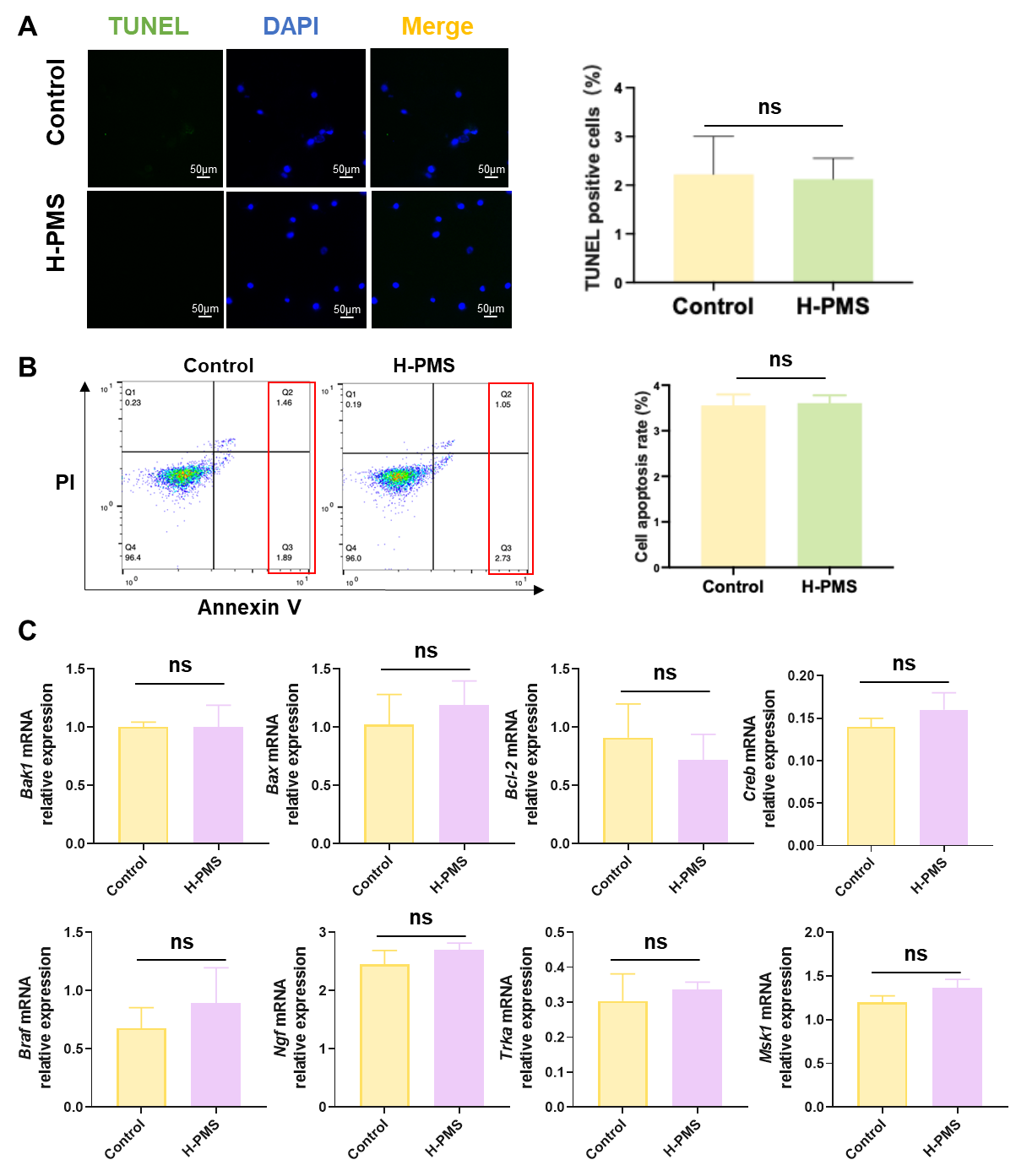
**

**Figure S4** (A)TUNEL staining revealed that PMS intervention did not significantly affect the TUNEL-positive cells; (B) Flow cytometry results revealed that PMS intervention did not significantly affect; (C) PCR results showed that the expression of *Bak1, Bax, Bcl-2, Creb, Braf, Ngf, Trka* and *Msk1* mRNA was did not significantly upregulated after PMS intervention. Cell groups included Control, and H-PMS.
